# ﻿Cell culture and karyotypic description of *Pseudophrynecoriacea* (Keferstein, 1868) (Amphibia, Anura) from the New South Wales Central Coast

**DOI:** 10.3897/compcytogen.17.113526

**Published:** 2023-11-10

**Authors:** Richard Mollard, Michael Mahony

**Affiliations:** 1 Melbourne Veterinary School, Faculty of Science, The University of Melbourne, Parkville, 3052, Australia The University of Melbourne Melbourne Australia; 2 Amphicell Pty Ltd, Cairns, Queensland, Australia Amphicell Pty Ltd Cairns Australia; 3 School of Environmental and Life Sciences, University of Newcastle, Callaghan, New South Wales, 2308, Australia University of Newcastle Callaghan Australia

**Keywords:** Cell culture, cryopreservation, karyotype, red-backed toadlet

## Abstract

The karyotype of the IUCN least concern red-backed toadlet Pseudophryne (P.) coriacea (Keferstein, 1868) from the New South Wales Central Coast is described following tissue culture of toe clipping macerates and conventional DAPI staining. The diploid number is 2n = 24. The karyotype is represented by six large and five small chromosomal pairs and one very small chromosomal pair. The very small chromosome 12 is 12% the size of chromosome 1. One of the large chromosomes is subtelocentric, two of the large chromosomes are submetacentric and the remaining chromosomes are metacentric. The putative nucleolus organiser region (NOR) is observed on chromosome 4. The diploid number and location of the putative NOR correlates to that of the previously published IUCN critically endangered *P.corroboree* (Moore 1953) and unpublished descriptions of the *P.coriacea* karyotype. This is the first described cell culture of a species from the genus *Pseudophryne* Fitzinger, 1843, first published analysis of the *P.coriacea* karyotype and the first published analysis of centromeric allocation of this genus. Globally there exists a large inventory of tissue samples in cryobanks that are not associated with known recovery mechanisms such as basic cell culture techniques. Detailed cytogenetic analyses of these cryobanked samples are therefore not possible. This work therefore enables: (i) a comparison of the *P.coriacea* karyotype with that of the critically endangered *P.corroboree* and (ii) a benchmark for repeat and future cytogenetic and genomic analyses of cryostored samples of this genus.

## ﻿Introduction

Recently documented amphibian declines resulting from disease and habitat destruction have placed nearly one third of all amphibian species at risk of extinction (Silla and Byrne 2019). Examples of animals at the forefront of this decline are *P.corroboree* (the southern corroboree frog) and *P.pengilleyi* (Wells and Wellington 1985), both critically endangered species for which restorative husbandry programs are required and cryobanking proposed (Morgan et al. 2008; Kouba et al. 2013; McFadden et al. 2013; Clulow and Clulow 2016; Skerratt et al. 2016; Rojahn et al. 2018). To date, captive breeding programs have demonstrated some success towards the long-term reintroduction of these animals into the wild (McFadden et al. 2013; Silla et al. 2018). No examples of successful cell culture with or without cryobanking to provide a non-invasive technique for long term auxiliary and repeat genomic monitoring or assisted reproduction programs, however, have been reported for any representative of this genus.

The red-backed toadlet *P.coriacea*, an IUCN least concern listed species, is endemic to the east coast and ranges of Australia, north of Sydney to southern Queensland (White 1993; Donnellan et al. 2012). The genus *Pseudophryne* Fitzinger, 1843 comprises 14 known species (O’Brien et al. 2018) for which the karyotype of only *P.corroboree* has been published (Mahony and Robinson 1986). Reports from unpublished data (Morescalchi and Ingram 1974; Mahony and Robinson 1986) suggest a highly conserved 2n = 24 karyotype across nine species in this genus, including *P.coriacea*, with an NOR also highly conserved on chromosome set 4. Centromeric positions remain to be described for any species in this genus. Detailed karyotypic information available for interspecies comparisons within this genus and associated information for assistance in conservation management programs are therefore wanting (Mahony and Robinson 1986; Potter and Deakin 2018).

This report serves four aims: (1) to demonstrate successful cultivation, passaging and cryopreservation of cells from *P.coriacea*, (2) to formally describe their karyotype including centromeric positions and NOR locations, (3) to facilitate future genetic comparisons for conservation management programs of species within this genus, and (4) provide a tissue resource for future cytogenetic and genomic work that would not require harming living animals.

## ﻿Material and methods

### ﻿Ethics

Relevant Australian State governmental and institutional ethics, licenses and permissions were obtained and the described research was conducted in accordance with The Code of Ethics of the World Medical Association of The Declaration of Helsinki and in compliance with the EU Directive 2010/63/EU for animal experiments. The animal specimen was collected by Michael Mahony under New South Wales National Parks Scientific Licence SL00190.

### ﻿Tissue culture and cryopreservation

Toe clippings obtained from an unsexed and deceased *P.coriacea* toadlet, euthanised for alternative research purposes, were prepared for culture and karyotyping according to previously described and detailed methods (Mollard 2018; Mollard et al. 2018; Bui-Marinos et al. 2022). Tissue was first rinsed in 70% v/v ethanol (Sigma Aldrich) and then washed consecutively three times in 0.22 μM (Merck Millipore) syringe (Terumo) filtered Amphibian Ringer’s Solution (AR; Cold Spring Harbor Protocols) at 4 °C. Tissues were macerated with fine scissors (Solingen) and transferred to 24 well plates (Falcon Multiwell™; GIBCO) containing preequilibrated Dulbecco’s Modified Eagle Medium (DMEM; Thermo Fisher Scientific) at room temperature (26 °C) in a 5% CO_2_/ 95% air atmospheric incubator (Steri-Cycle CO_2_ Incubator; FORMA) according to previously described methods (Speare and Smith 1992; Fukui et al. 2003; Ferris et al. 2010; Strauß et al. 2011) with gentamicin replaced with 1000 units/mL penicillin (Sigma Aldrich) and 1000 μg/mL streptomycin (Sigma Aldrich). After four days, when individual cells could be observed to emerge from the tissue pieces, one half of the media was changed daily for three days and thereafter the entire media was changed every one to two days. For passaging, cultures were rinsed with AR and adherent cells were trypsinised with 0.25% trypsin/0.02% ethylenediaminetetraacetic acid solution (Sigma Aldrich) and replated at a 1:3 dilution. For cryopreservation, following trypsinisation, cells were resuspended in 100 μl of culture DMEM containing 10% dimethyl sulfoxide (DMSO; Sigma Aldrich) in 1 ml cryotubes (Nunc®), placed at minus 80 °C (CSK Group) overnight and then transferred to liquid nitrogen for storage (Taylor Wharton). For culture following cryopreservation, cryovials were placed on ice until the medium was visibly thawed. DMSO in the cryovial was diluted to 0.5% with DMEM culture media and the media was then transferred to one well of a 24 well plate for reseeding. The medium was changed with fresh culture medium after 48 hours and then daily until karyotyping. Cells were photographed periodically using an Olympus IX70 – S8F2 inverted microscope, a ProgRes®C3 (Jenoptik, Germany) camera and ProRes® CapturePro Software Version 2.8.8.

### ﻿Karyotyping

Karyotyping was performed according to modifications of previously described techniques (Howe et al. 2014). When culture wells had reached approximately 70% confluency, cells were treated for six hours with 0.1 μg/ml KaryoMAX® colcemid (GIBCO), removed from the culture dish with a two minute trypsin treatment, incubated in hypotonic 0.027 M Na_3_Citrate (Sigma Aldrich) for five minutes, and fixed in Carnoy’s fixative (Cold Spring Harbor) overnight at 4 °C. Microdrops were released from a 20 μl Gilson pipette onto ethanol cleaned glass microscope slides (Thermo Fisher Scientific) held at an approximately 45 degree angle from a height of approximately 20 cm, and above a water bath (Sigma Aldrich) preheated to 80 °C. Karyotype preparations were airdried overnight in a dust free environment. Spread cells were then stained with 4’,6-diamino-2-phenylindole (DAPI; 500 ng/ml; Sigma Aldrich) and coverslipped (Menzel-Gläser) under Gelvatol mounting medium (Cold Spring Harbor Protocols). For numbering chromosomes, the largest chromosome was designated chromosome 1 and the remainder were designated in descending size order. Image J software with the Levan plugin (Levan et al. 1964) was used to measure chromosomal arm lengths. Metacentric, submetacentric and subtelocentric chromosomal designation were defined as a long arm to short arm ratios of 1–1.69, 1.7–2.99 and 3–6.99, respectively (Levan et al. 1964). Images were captured at 1000 × with an Olympus BX60 microscope, colour CCD Leica DFC425C camera, EL-6000 Leica light source and Leica LAS-AF and QCapture Pro7 Version 7.0.5 Build 4325 software (QImaging Inc, USA).

## ﻿Results and discussion

**Figure 1.**
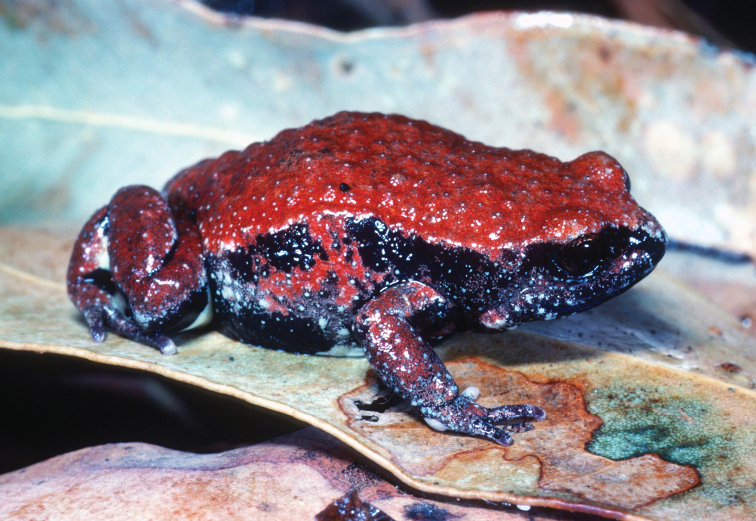
*Pseudophrynecoriacea*. Photographed by Michael Mahony at Wallingat State Forrest, New South Wales, Australia, 1982.

**Figure 2.**
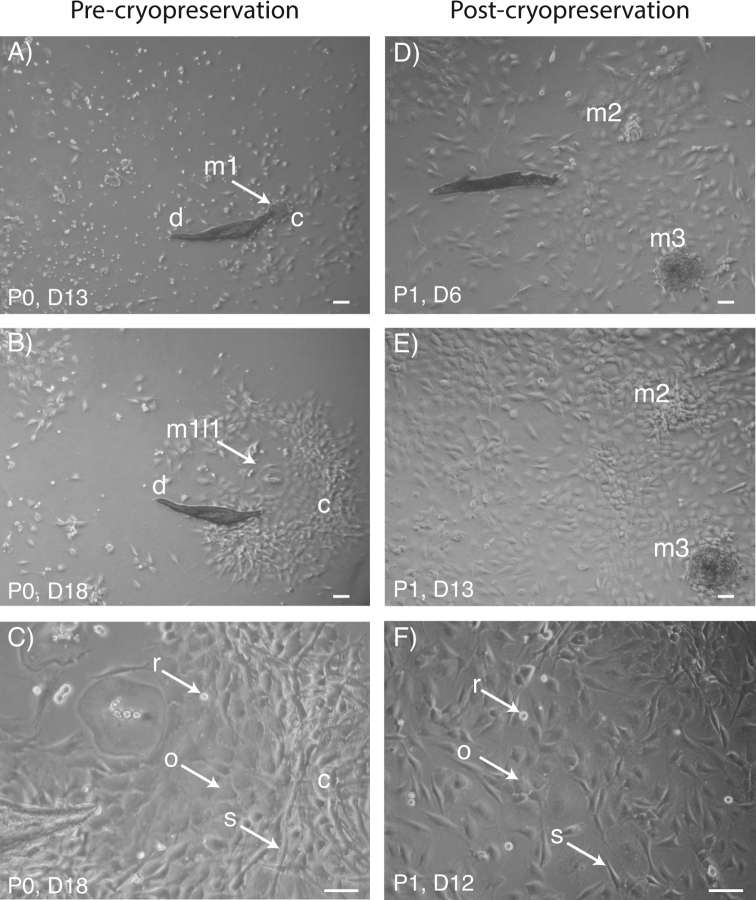
*Pseudophrynecoriacea* macerated and cultured toe clippings **A–C** primary culture prior to cryopreservation (P0) **D–F** passage 1 cells, post cryopreservation (P1) **A, B, D, E** low power **C, F** high power **A, B** P0 cells form an expanding cluster (c) adjacent to the reference debris (d) at days 13 (D13) and D18 **C** P0 cells are either ovoid/polygonal (o) or spindle-shaped (s); rounded cells (r) are also observed **D, E** post-cryopreservation, P1 cells form two expanding mass reference points (m2 and m3) to reach approximately 70% confluency by D13 **F** post-cryopreservation P1 cells are both ovoid/polygonal (o) and spindle-shaped (s); rounded cells (r) are also observed. Scale bars: 10 μM.

Toe macerates from an unsexed *P.coriacea* were placed in culture and individual cells were observed as attached single cells or within small expanding cell masses during the following two weeks (Figs 1, 2). Under high power inverted phase contrast microscopy, and at day 18 (D18), a mixed cell population comprising spindle-shaped and ovoid/polygonal morphology was observed. Rounded/semi-detached cells were presumed to be mitotic cells. At D18 and approximately 40% confluency, cells were trypsinised and cryopreserved in liquid nitrogen.

Following a 12 month period of cryopreservation, cells were thawed into two separate wells of a 24 well plate. Passage 1 (P1) cells post-thawing attached within 48 hours as both cell clumps and single cells, and formed colony outgrowths resulting in approximate 70% confluency by D13 (Fig. 1). Mixed spindle-shaped and ovoid/polygonal cell populations were observed, as well as more condensed rounded cells characterizing a presumed mitotic phenotype. Cells from one dish were processed for karyotyping, while cells from the second dish were passaged and subsequently processed for cryopreservation. A total of 200 000 passage two *P.coriacea* cells were cryopreserved in a seven week total culture period.

**Figure 3.**
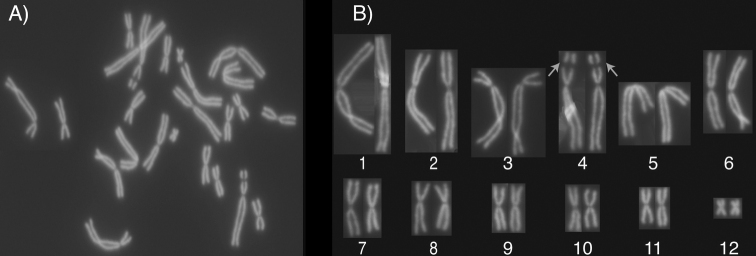
*Pseudophrynecoriacea* karyotype **A** metaphase spread and **B** chromosomal pairs arranged in descending order relative to size and aligned by centromeric position. A 2N = 24 diploid chromosome number and the presence of a DAPI negative region on each of the short arms of chromosome 4 (arrows) are evident. Chromosomes 1 to 6 are larger, whereas chromosomes 7 to 11 are smaller in size, and chromosome 12 is smaller still.

Of the first 27 metaphase *P.coriacea* chromosome spreads identified and counted, 26 displayed a 2N = 24 chromosomal count and one displayed a chromosomal count of 15, with the latter a probable artefact of the cell spreading technique (24_incidence_ = 96%; Fig. 3A). Six metaphase spreads were arranged in descending order of size to identify six large and five small chromosomal pairs and one very small chromosomal pair (Fig. 3B). A DAPI negative region was observed on the short arms of each chromosome pair number four (representing a presumptive NOR). Chromosomes 1, 2, 6, 7, 8, 9, 10, 11 and 12 are apparently metacentric, chromosomes 4 and 5 are apparently submetacentric and chromosome 3 is apparently subtelocentric (Table 1). Chromosome lengths were calculated relative to chromosome 1, not including measurements of the secondary restrictions on chromosome 4 (Table 1). The notably smaller chromosome 12 is 12% the size of chromosome 1.

**Table 1.** Chromosome log arm to short arm ratios with centromeric designations and overall relative lengths. The long arm to short arm ratios are provided from the average of six prepared and measured karyotypes +/- standard deviation. Relative lengths are provided from the percentage sum of each allocated and corresponding chromosomal set from the six individual karyotypes. The relative chromosome 5 length is smaller than relative chromosome 4 length at only four decimal places. Of note, inclusion of the secondary restriction measurement places chromosome 4 as chromosome 3, with a relative length of 0.7262.

	**Chromosome number**					
	**1**	**2**	**3**	**4**	**5**	**6**
Arm ratios	1.23 ± 0.1	1.62 ± 0.3	3.9 ± 0.6	2.15 ± 0.4	1.95 ± 0.2	1.27 ± 0.2
Designation	Metacentric	Metacentric	Subtelocentric	Submetacentric	Submetacentric	Metacentric
Relative length	1	0.7793	0.7104	0.7046	0.7043	0.6311
	**Chromosome number**					
	**7**	**8**	**9**	**10**	**11**	**12**
Arm ratios	1.57 ± 0.4	1.35 ± 0.3	1.30 ± 0.2	1.37 ± 0.2	1.3 ± 0.2	1.28 ± 0.3
Designation	Metacentric	Metacentric	Metacentric	Metacentric	Metacentric	Metacentric
Relative length	0.3941	0.3762	0.3365	0.3101	0.2823	0.1175

While sperm cryobanking techniques have made significant advancements, methods for the cryopreservation of oocytes, embryos or amphibians suitable for conservation programs have not (Browne et al. 2019; Burger et al. 2022; Lampert et al. 2022). The cryostorage of karyotypically stable diploid nuclei amenable to recovery therefore represents a near term and important process for genomic and cytogenetic work and an additional resource for future conservation related assisted reproductive technologies (Kouba et al. 2013; Clulow and Clulow 2016; Zimkus et al. 2018).

## ﻿Conclusion

The overall *P.coriacea* karyotype with 2n = 24 and the location of the presumptive NOR on chromosome 4 agrees with previously unpublished reports for this species (Morescalchi and Ingram 1978; Mahony and Robinson 1980). It is also consistent with that described for *P.corroboree* (Mahony and Robinson 1980) and the unpublished data (Morescalchi and Ingram 1978; Mahony and Robinson 1980) on a further seven species from this genus including *P.pengilleyi*. The description of the centromeric positions and relative lengths for any species of this genus is novel. This study serves as a prototype for future comparisons of centromeric descriptions and karyotypes of species from the genus *Pseudophryne*, thus aiding conservation management programs.

## ﻿Competing Interests

Richard Mollard has registered a company called Amphicell Pty Ltd (www.amphicell.com). Amphicell Pty Ltd received no funding for this work and privately provided the materials to execute the experimental procedures described in this study.
